# Numerical model for enhancing stimulated Brillouin scattering in optical microfibers

**DOI:** 10.12688/f1000research.51029.1

**Published:** 2021-06-30

**Authors:** Soon Heng Yeap, Siamak Dawazdah Emami, Hairul Azhar Abdul-Rashid

**Affiliations:** 1 Research Department, Broadcom, Bayan Lepas, Penang, 87300, Malaysia; 2 Fiber Optics Research Center, Multimedia University, Cyberjaya, Selangor, 63100, Malaysia; 3 Laser and Plasma Research Institute, Shahid Beheshti University, Evin, Tehran, Iran

**Keywords:** Stimulated Brillouin Scattering, Brillouin Shift Frequency, Effective Refractive Index, COMSOL, Slow Light

## Abstract

Stimulated Brillouin scattering (SBS) is useful, among others for generating slow light, sensing and amplification. SBS was previously viewed as a poor method due to the limitation on optical power in high-powered photonic applications. However, considering the many possible applications using SBS, it is now of interest to enhance SBS in areas of Brillouin frequency shift together with Brillouin Gain. A numerical model, using a fully vectorial approach, by employing the finite element method, was developed to investigate methods for enhancing SBS in optical fiber. This paper describes the method related to the numerical model and discusses the analysis between the interactions of horizontal, shear and hybrid acoustic modes; and optical modes in optical fiber. Two case studies were used to demonstrate this. Based on this numerical model, we report the influence of core radius, clad radius and effective refractive index on the Brillouin frequency shift and gain. We observe the difference of Brillouin shift frequency between a normal silica optical fiber and that of a tapered fiber where nonlinearities are higher. Also observed, the different core radii used and their respective Brillouin shift. For future work, the COMSOL model can also be used for the following areas of research, including simulating “surface Brillouin shift” and also to provide in-sights to the Brillouin shift frequency vB of various structures of waveguides, e.g circular, and triangular, and also to examine specialty fibers, e.g. Thulium and Chalcogenide doped fibers, and their effects on Brillouin shift frequency.

## Introduction

Stimulated Brillouin scattering (SBS) is a nonlinear process caused by acoustic phonon scattering propagating in the backward direction. Acoustic vibration across mediums scatters the incident light of the pump wave causing an acoustic frequency shift resulting in Stokes and anti-Stokes waves. The process of transferring energy from the pump wave to the Stokes wave is known as the scattering phenomenon. The Stokes wave counter propagates in the opposite direction to the pump wave. The presence of acoustic waves propagating on the medium’s surface is known as the surface acoustic wave. SBS theory was first explained by Leon Brillouin in 1922, and since then related experimental works have been performed in the past decade. ^1^ This paper describes a numerical model that analyzes the interactions of horizontal, shear and hybrid acoustic modes; and optical modes in optical fibers. The numerical model was developed using COMSOL Multiphysics. Two case studies were used to demonstrate the model’s utility. Based on this numerical model, we report the influence of core radius, clad radius and effective refractive index on the Brillouin frequency shift and gain coefficient.

## Literature review

Prior studies related to SBS have focused on the acoustic frequency shift of different types of fibers. In theory, peak Brillouin gain for a standard silica fiber is approximated to be 5 × 10 ^−11^ m/W. ^2^ In addition, fibers around 2.6 × 10 ^−12^ m/W have been presented by Nikles *et al*. ^3^ Optimized SBS acoustic frequency shift for tellurite photonic crystal fiber (PCF) was recorded at 8.43 GHz, which gives 9.48 × 10 ^−11^ m/W of Brillouin gain. ^4^ Experimental results for SBS across a chalcogenide fiber was demonstrated by Song *et al.* at 6.08 × 10 ^−9^ m/W, showing a higher Brillouin gain compared to a standard silica fiber. ^5^ Woodward *et al.* reported an experimental study on SBS in small-core PCF, ^6^ which discussed the complexity of acoustic wave dynamics for different wavelengths of light, overlap between optical waves were minimum at 532 nm. Consequently, at 1550 nm, a higher overlap is achieved, which contributes to a higher Brillouin gain, and therefore a lower threshold power of 1160 mW. ^6^ More recently, Tchahame *et al.* demonstrated a multimode Brillouin spectrum across a long tapered PCF. ^7^ Beugnot *et al.* successfully conducted both the first numerical and experimental work on surface acoustic waves (SAW) of silica microfiber in 2014, with a Brillouin gain equivalent to 1.4 × 10 ^−12^ m/W. ^8^ The idea is greatly appreciated as SAW has outstanding potential contribution in the optical sensor due to its sensitivity on various physical perturbations. However, the limitation is that threshold power of the tapered fiber is relatively high and so it is not feasible in optical application. Acoustic confinement in the fiber core is required to ensure high overlap with the optic wave. Cherif *et al.* studied the Brillouin spectrum of SBS characterization for small core tellurite PCF with variation in air-filling ratio. ^4^ In addition, previous results by Hu *et al.* showed a low threshold power of 52 mW on chalcogenide fibers whose acoustic waves were confined to the core. ^9^ However, for SBS across tapered silica fibers an undesired high threshold power was observed due to a reduced interaction of the surface acoustic wave with the optical wave. To counter this effect, gold and silver cladding materials have been proposed. The finding from Kim *et al.* shows novel shear Brillouin scattering detection using microscopic resolution. ^10^ This finding is fundamental as previous experimental set ups focused on longitudinal acoustic wave propagation.

Several experimental studies have been carried out by researchers to study the behavior of SBS in optical fibers. However, there are several limitations in experimental studies such as fabrication certainty, environmental influences and access to laboratory equipment. An accurate modeling tool, developed for the purpose of aiding experimental studies, would be beneficial to speed up research while preserving a good level of accuracy and confidence. Finite Element Method (FEM), a method that applies meshing technique to solve partial differential equations (PDE) with a certain boundary condition. ^14^ FEM received great attention when improvements in handling boundary conditions with the implication of penalty functions was discussed. ^15^ This gave FEM the preference in optical fiber modeling considering the boundary issue as the problem that other methods fail in. Ham et al. ^16^ then introduced the complete numerical solution, where FEM is used with spectral method that provides accuracy and consistency for 2-D and 3-D cases involving harmonic functions. These findings made FEM more suitable for optical fiber numerical modeling. Sriratanavaree et al. ^17^ used FEM in the study of optical and acoustic wave interaction in silicon slot waveguides. Subsequently, Monfared et al. ^18^ showed how FEM modeled for composite behavior of bond particle at fiber interface. Findings from Liu et al. ^19^ showed a numerical solution using FEM to enumerate the tension and bending in optical fiber accurately. A report on optical fiber modeling and simulation of effective refractive index for tapered fiber with finite element method was deliberated by Lee et al. ^20^ Rahman et al. ^11, 12^ reported numerical modeling of SBS, considering the optical fundamental mode in the optical fiber using FEM.

## Methods

We present a numerical model to optimize Brillouin frequency shift and gain based on various core diameters of the tapered region of silica microfiber structures. To further understand the interactions, the COMSOL model should be capable of modelling solutions in the given structures of the optical fiber. Previous research was mainly done to enhance performance on spatial resolution and also sensing range, but there have not been many insights for gain. The ability to increase the Brillouin gain coefficient has opened new opportunities to control their interaction, and several new industrial and commercial applications. In this research work, we demonstrate the numerical model for a microfiber design that is expected to increase Brillouin gain coefficient. The fully vectorial method, developed in COMSOL, is used in this case to determine the contributions of various optical fibre parameters towards SBS, thus aiding the design of microfibers with enhanced SBS performance. The equation below is used for the analysis of light guidance where H is the full vectorial magnetic field, * represents the complex conjugate and transpose, ω ^2^ denotes the eigenvalue where ω is the optical angular frequency of the wave and ε and μ are the permittivity and permeability, respectively. ^21^

$$\omega^{2}=\frac{\int \left({\left(\nabla \times H\right)}^{*}.\in^{-1}\left(\nabla \times H\right)+\rho \left(\nabla .H\right)*\left(\nabla .H\right)\right)\;\text{dxdy}}{\int H^{*}\mathrm{\mu H}\;\text{dxdy}}\;$$ (1)

Equation (1) solves for the propagation constant of optical modes in optical waveguides, which can also guide acoustic mode. The propagation constant of the optical wave, *β*, is defined as *β _optic_* = 2 *π*n _eff_/ *λ.* There are two basic types of acoustic waves, namely shear and longitudinal acoustic waves. Shear waves are associated with dominant material dispersion in the transverse directions, which is perpendicular to the direction of propagation, taken here as the z-axis. On the other hand, for a longitudinal wave, expansion and contraction of the wave is associated with particle movements along the z direction which is in parallel to the wave propagation. However, acoustic wave propagating through a waveguide can be a combination of shear and longitudinal acoustic waves. Brillouin frequency shift of the Stokes wave is given as f = 2n *_eff_* V *_ac_*/ *λ*, where, V *_ac_* is the acoustic velocity. The acoustic wave satisfies Hooke’s Law ^12^ which relates to the stress (tensor) and strain (force) of the waveguiding materials. Electric field associated with a high power optical signal causes molecular movements due to electrostriction process. ^13^ Such a material movement can generate acoustic waves that leads to density variation along the waveguide. The time and space dependent density variation changes the refractive index profile and produces a moving optical grating. This grating can reflect incoming light when its wavelength matches the spatial period of the gratings generated by the acoustic wave. Above a threshold power, if phase matching conditions are satisfied, it can inhibit forward guidance of the incoming light. The backward scattered reflected wave is frequency shifted, which explains the occurrence of the Stokes Wave. The relationship between optic and acoustic propagation for phase matching condition can be given as: K *_acoustic_* = 2 *β _optic_* where K *_acoustic_* is the acoustic propagation constant and this will be double of the *β _optic_*, the optic propagation constant.

For the SBS characterization in the optical fiber, both its guided optical and acoustic modes can be obtained using the FEM. The n _eff_ for the optical mode in a fibre for a given radius is first calculated using H-field based FEM model. Eigenvector and eigenvalue of acoustic waves are also obtained and then the acoustic mode patterns are generated. At phase matched conditions, the acoustic wave propagation constant is double the value of the optical wave propagation constant: K _acoustic_ = 2 *β* optic.

In this research work, the fully vectorial approach was used to solve the optical wave equations for *n _eff_* using the commercial COMSOL software. The optical parameters E(x,y) and *n _eff_*, are obtained by solving the equation below:

$$\Delta_{t}^{2}+\left(\frac{2\pi }{\lambda }\right)2\left(n^{2}-n_{eff}^{2}\right)E=0$$ (2)

Where ∆t is transverse Laplacian operator in the ( *x*, *y*) direction, while *n*_*eff*_ is the effective refractive index of fundamental optical mode, ^9^ directly related to Brillouin frequency shift via the Bragg condition. ^10^ The acoustic wave, which consists of the stress and strain components, are governed by Hooke’s Law ^12^ whereby solving the equation below would yield its displacement.

$$T_{ij}=c_{ijkl}S_{kl};\;\;i,j,kl=x,y,z$$ (3)

where *T* denotes the stress field and S represents the force field which is equivalent to partial differentiation of displacement. c _ijkl_ is the tensor relation of elastic stiffness where *i, j, kl* are equivalent to propagation in *x*, *y* and *z* direction respectively. ^22^ For an isotropic medium with uniform wave propagation, the elastic stiffness constant is given by the longitudinal and shear velocity that is dependent on the material properties of the optical fiber core and cladding. ^12^

For the purpose of calculating the Brillouin gain, the overlap factor from the fully vectorial approach was used. The overlap between optical wave and acoustic wave is given in equation (4) below. ^21^

$$\mathrm{\Gamma ij}=\frac{\int {\left[\left|H_{i}^{2}\left(x,y\right)\right|\left|U_{j}\left(x,y\right)\right|\right]}^{2}\mathrm{dA}}{\int {\left|H_{i}\left(x,y\right)\right|}^{4}\mathrm{dA}\int {\left|U_{j}\left(x,y\right)\right|}^{2}\mathrm{dA}}\;$$ (4)

where *H _i_* ( *x, y*) is the fundamental mode in optical wave and *U _j_* ( *x, y*) is the displacement vector of acoustic wave. Optic-acoustic wave overlap factor is influenced by the acoustic wave’s strain field and refractive index of the optical fiber. ^11^ Both the optical and acoustic wave vectors have to be normalized to calculate the overlap factor.

The Brillouin gain coefficient is represented by the equation below: ^23^

$$g_{B}\left(v\right)=g_{p}=\frac{4\pi^{3}{n_{\mathrm{eff}}}^{8}{p^{2}}_{12}}{c\lambda_{p}^{3}\rho_{0}v_{B}\Delta v_{B}}\;$$ (5)

Where *ρ*_0_ is fiber core density, *p*_12_ is elasto-optic coefficient which contributes to the periodic light scattering and is FWHM of acoustic wave in SBS. ^23^

### COMSOL parameters

**Method in COMSOL**

Based on the parameters in Table 1, SBS characterization using the fully vectorial approach was performed on various core diameters of the tapered region of the silica microfiber and was verified against earlier results by H. J. Lee. ^23^ The n *_eff_* were calculated for all core diameters. Refractive index for the core and clad used were 1.4502 and 1.445 respectively considering the measured values in a typical single mode fiber. ^21^ Following that, three cases of acoustic wave, shear, longitudinal and hybrid behavior were analyzed.

**Table 1.** Tapered fiber parameters.

Case study	Core (diameter)	Cladding (diameter)	Refractive Index (core/clad)	Density of material *ρ* (kg/m ^3^)	Wavelength
1	2 *μ*m	6 *μ*m	1.4502/1.445	2202	1550 nm
2	4 *μ*m	12 *μ*m			

***Optical mode solver***

To obtain the *n _eff_* results for the two case studies covered in this research, the optical mode equation was solved using the COMSOL RF module. In the RF module the electromagnetic waves, frequency domain physics engine was used. The geometric structures of the fibers were generated using the two dimensional space dimension. From there, the parameters as denoted in Table 1 were entered into the solver. The density, refractive index of both the core and clad values are declared under the materials section. Thereafter, the mode analysis frequency is set to the desired wavelength of 1550 nm and the perfect electrical conductor boundary condition was used. As with all FEM solvers meshing is required. For this case, the triangular mesh and the “finer” element size was used. The effective mode index or *n _eff_* for the fundamental mode can thereafter be obtained after computing the solver.

***Acoustic mode solver***

As for the acoustic model, the acoustic waves of shear and longitudinal were evaluated independently to record the findings. Thereafter, the hybrid acoustic wave across the fiber in which both shear and longitudinal acoustic waves co-exist were examined. The hybrid mode model was the model used to determine the Brillouin Shift Frequency $v_{B}$ as both these waves propagate together in real life conditions. Table 2 shows the velocity assigned for core clad region in each respective case. In the case of pure shear acoustic, longitudinal velocity for the core region were made equivalent to 5736 m/s, this is to prevent interruption of longitudinal acoustic wave. Similarly, for pure longitudinal acoustic, the shear acoustic of the core was made to 3625 m/s. For the hybrid acoustic wave, the velocities of both the longitudinal and shear waves of the clad are defined to be slightly higher than the core. ^21^ This is to prevent interruption of acoustic wave between the two regions. The core-clad ratio was taken to be 1:3 so that the clad region is long enough to prevent wave reflection from the outer side of clad back to the inner core. Equation 3 being a partial differential equation is solved using the Mathematics physics engine. The weak form PDE interface was used to solve equation 3. Like the optical model, the geometric structures were setup and defined in the model and the values for the acoustic wave for both shear and longitudinal velocities as tabulated in Table 2 were used. The Drichelet boundary condition was used for acoustic model simulation and the mesh setting for the acoustic model is similar with the optical model whereby the triangular mesh was used with the “finer” element size used. Computing the solver would return multiple results of eigenvalues. The eigenvalue returning the fundamental mode plots would be the one selected as the Brillouin Shift Frequency $v_{B}$ result.

**Table 2.** Acoustic velocity parameters for acoustic model simulation. ^21^

Case	Region	V _***l***_ (m/s)	V _***s***_ (m/s)
Shear acoustic	Core	5933	3625
Shear acoustic	Clad	5933	3764
Longitudinal acoustic	Core	5736	3764
Longitudinal acoustic	Clad	5933	3764
Hybrid acoustic	Core	5933	3625
Hybrid acoustic	Clad	5933	3764

## Results and discussion

**Figure 1.**
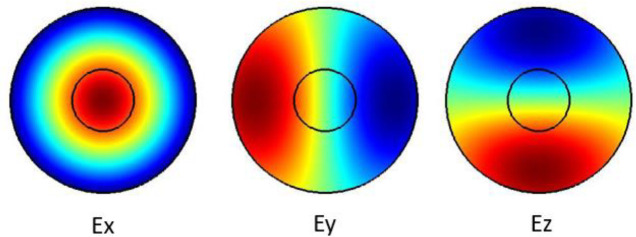
Case study 1: optical results.

The plots in Figure 1 show the Ex, Ey and the Ez components for the optical modes. Based on the numerical model, the *n _eff_* was found to be 1.431599.

**Figure 2.**
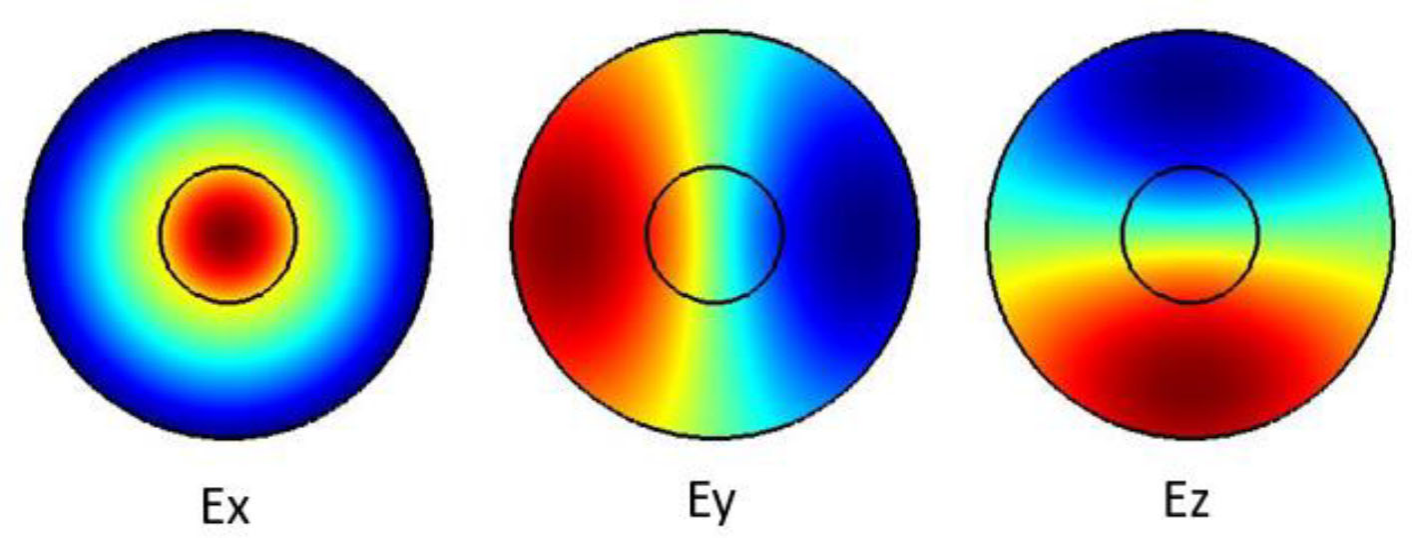
Case study 2: optical results.

The plots in Figure 2 show the Ex, Ey and the Ez components for the optical modes respectively. Based on the numerical model, the *n _eff_* was found to be 1.441802

**Figure 3.**
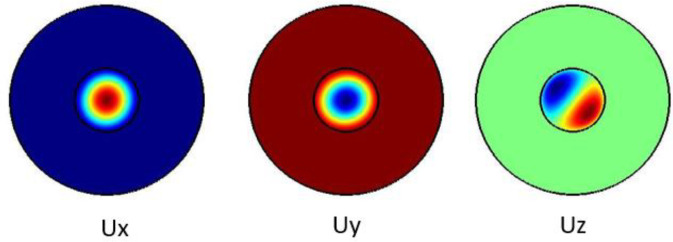
Case study 1: acoustic results for shear modes.

Figure 3 shows the pure shear acoustic mode along the silica microfiber where it dominates along x direction. For shear acoustic mode propagation, frequency shift is at 6.61 GHz with acoustic velocity of 3578 m/s.

**Figure 4.**
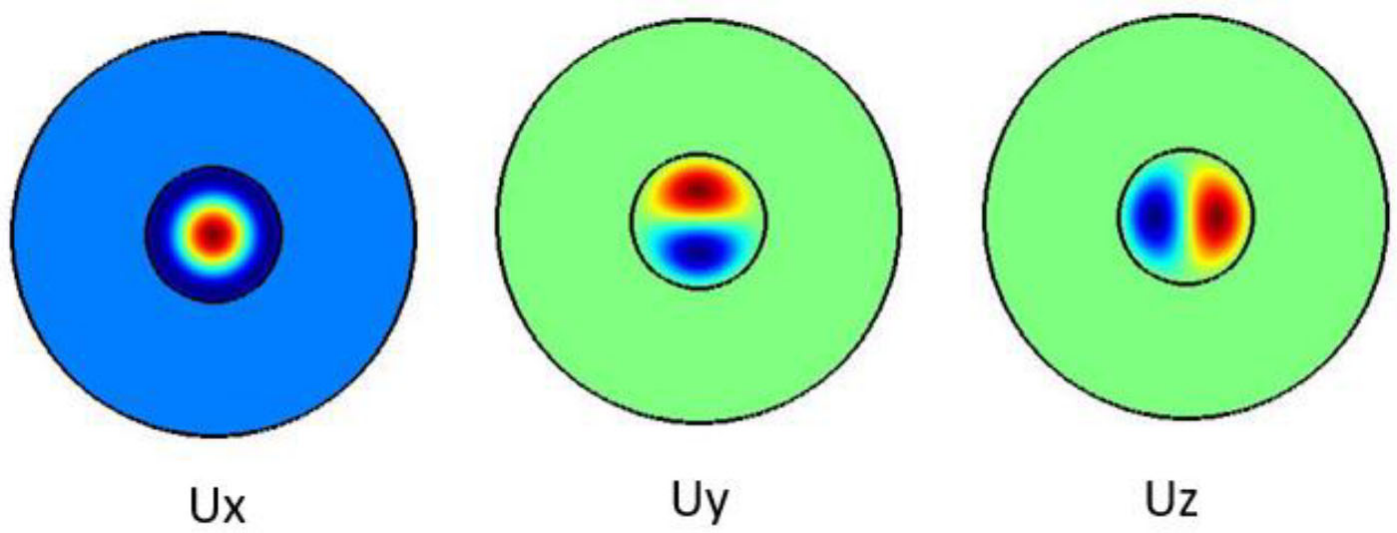
Case study 2: acoustic results for shear modes.

Figure 4 shows the pure shear acoustic mode along the silica microfiber where it dominates along x direction. For shear acoustic mode propagation, frequency shift is at 6.637 GHz with acoustic velocity of 3567 m/s.

**Figure 5.**
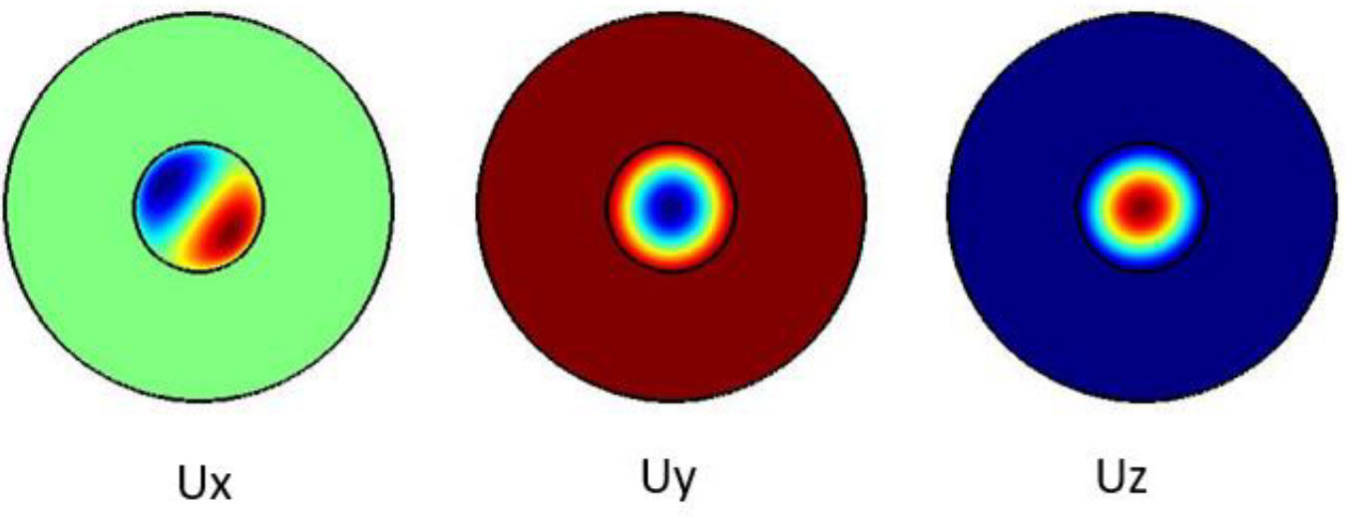
Case study 1: acoustic results for longitudinal modes.

Figure 5 shows the pure longitudinal acoustic mode along the silica microfiber where it dominates along z direction. For longitudinal acoustic mode propagation, frequency shift is at 6.634 GHz with acoustic velocity of 3591 m/s.

**Figure 6.**
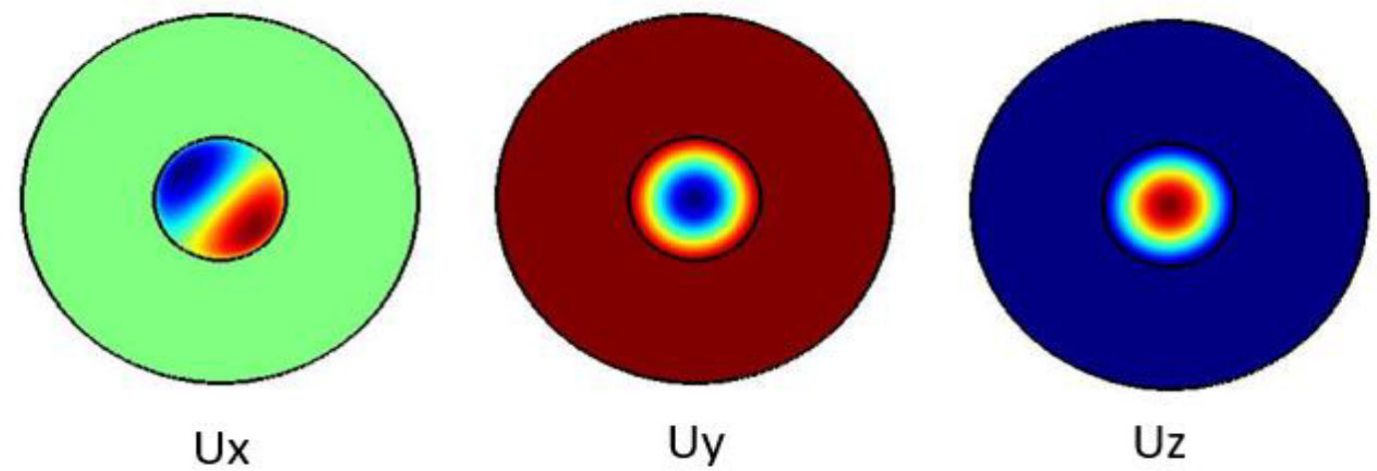
Case study 2: acoustic results for longitudinal modes.

Figure 6 shows the pure longitudinal acoustic mode along the silica microfiber where it dominates along z direction. For longitudinal acoustic mode propagation, frequency shift is at 6.639 GHz with acoustic velocity of 3568 m/s.

**Figure 7.**
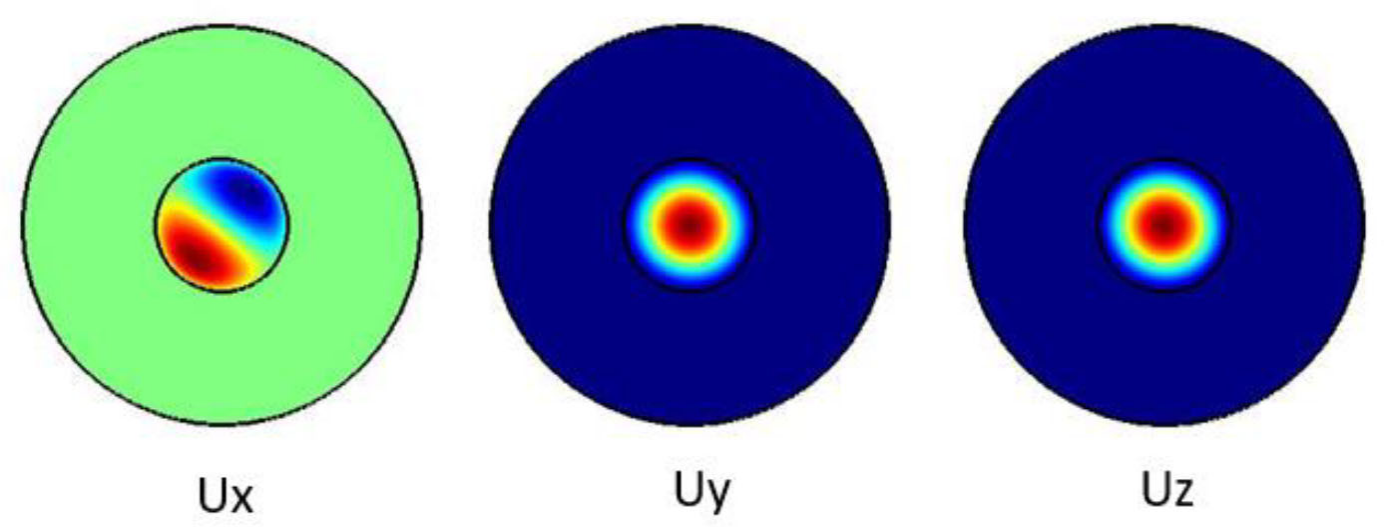
Case study 1: acoustic results for hybrid modes.

Figure 7 shows the hybrid acoustic mode along the silica microfiber where it dominates along z direction. For hybrid acoustic mode propagation, frequency shift is at 6.611 GHz with acoustic velocity of 3578 m/s.

**Figure 8.**
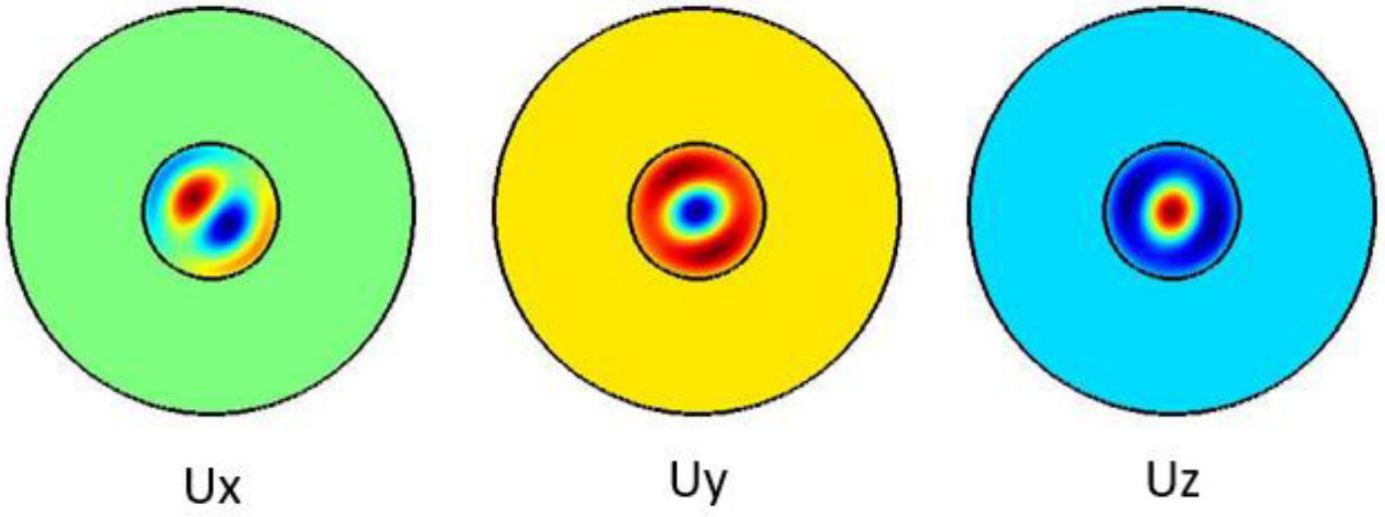
Case study 2: acoustic results for hybrid modes.

Figure 8 shows the hybrid acoustic mode along the silica microfiber where it dominates along z direction. For hybrid acoustic mode propagation, frequency shift is at 6.638 GHz with acoustic velocity of 3568 m/s.

From the two case studies simulated using the COMSOL numerical model, we find that the values of the effective refractive index generated by the optical mode model increases as the core and clad diameter increases The results are recorded in Table 3 below.

**Table 3.** Effective refractive index results.

Case study	Core diameter	Clad diameter	Effective refractive index
1	2 um	6 um	1.431599
2	4 um	12 um	1.441802

**Figure 9.**
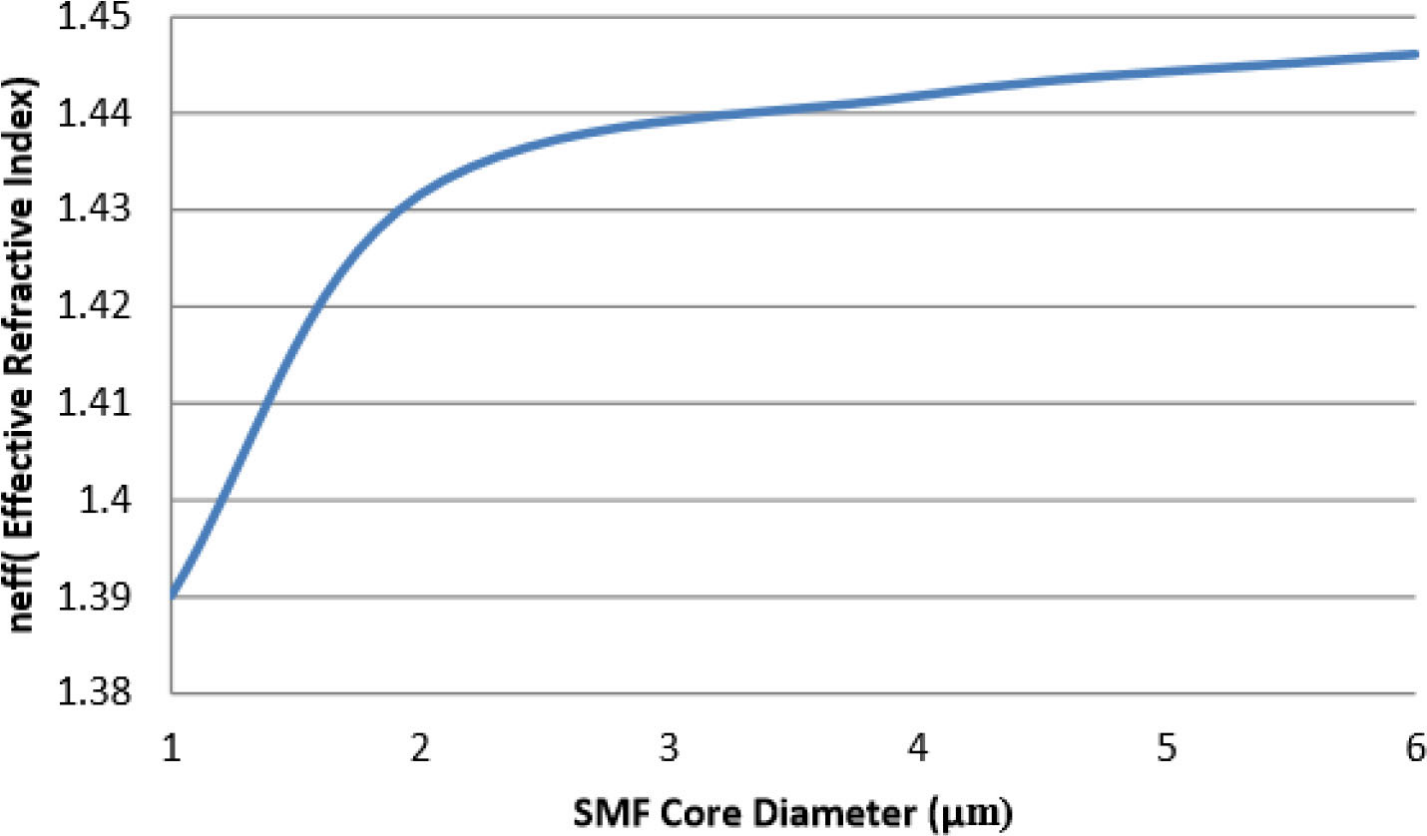
Relationship between *n _eff_* and silica microfiber diameter.

Figure 9 demonstrates the relationship between *n _eff_* and silica microfiber diameter. *n _eff_* in this case here can be seen increasing as the core diameter of optical fiber increases. The *n _eff_* value increases ranging from 1.390115 to 1.446122 for 1 μm to 6 μm core diameter of the optical fiber. The values are generated based on the optical model developed in COMSOL. The effective mode index *n _eff_* is based on the fundamental mode for these fibers.

From the results obtained and shown in Table 4 based on the acoustic model simulations, the smaller core and clad radius of an optical fiber will produce a lower Brillouin shift frequency compared to an optical fiber with a larger core and clad radii. As for the acoustic velocities observed in the optical fibers, a smaller core and clad radii produces a higher acoustic velocity. The Brillouin frequency shift is due to the optical-acoustic interaction. The overlap integral as stated in equation 4 demonstrates the overlap ratio between the acoustic and optical mode in the optical fiber. Shown below are the overlap ratios with respect to their core diameter. The overlap ratios are obtained by solving the overlap integral equation and are tabulated in Table 5.

**Table 4.** Brillouin shift frequency and acoustic velocity for shear, longitudinal and hybrid modes results.

Case study	1	2
Core diameter	2 *μ*m	4 *μ*m
Cladding diameter	6 *μ*m	12 *μ*m
Brillouin frequency shift for shear modes	6.610 GHz	6.637 GHz
Acoustic velocity for shear modes	3578 m/s	3567 m/s
Brillouin frequency shift for longitudinal modes	6.634 GHz	6.639 GHz
Acoustic velocity for longitudinal modes	3591 m/s	3568 m/s
Brillouin frequency shift for hybrid modes	6.611 GHz	6.638 GHz
Acoustic velocity for hybrid modes	3578 m/s	3568 m/s

**Table 5.** Overlap ratio.

Core diameter	Clad diameter	Overlap ratio
2 um	6 um	0.126
4 um	12 um	0.073

From the results in Table 5, it can be seen that the overlap ratio decreases as the core diameter increases.

The Brillouin gain coefficient values can be calculated by substituting the values obtained from the numerical model into the equation as denoted by Equation 5. Table 6 shows the Brillouin gain coefficient for the 2 optical fibers that were modeled.

**Table 6.** Brillouin gain.

Core diameter	Clad diameter	Brillouin gain
2 um	6 um	1.780 × 10 ^−11^ m/W
4 um	12 um	3.149 × 10 ^−12^ m/W

## Conclusion

The main aim of this research is to investigate the Brillouin shift in a tapered silica fiber of different core and clad radii by stimulated Brillouin scattering. To do that, numerical model simulations were developed to study the behavior of the optical wave and acoustic wave propagation in the tapered fiber region. The study on the acoustic wave, which was further divided into three other waves, namely the shear, longitudinal and hybrid mode were evaluated. The hybrid mode would produce the Brillouin shift $v_{B}$ which this research is interested in. As the SBS phenomenon benefits from the nonlinearities of a fiber, the present research documents the difference of Brillouin shift frequency between two different core and clad diameters of the tapered fiber region. The Brillouin Frequency Shift $v_{B}$ occurs at lower frequencies for a tapered fiber with smaller core and cladding dimensions. Nonlinear effects were also observed to be higher in a smaller diameter fiber based on the overlap factor and Brillouin gain reported. For future work, this COMSOL model can also be used for the following areas of research which includes simulating “Surface Brillouin Shift” and also to provide in-sights to the Brillouin shift frequency $v_{B}$ of various structures of waveguides, e.g. circular and triangular, and also to examine specialty fibers, e.g. Thulium and Chalcogenide, and their effects on the Brillouin shift frequency.

## Data availability

### Underlying data

DRYAD: Dataset of Numerical Model For Enhancing Stimulated Brillouin Scattering In Optical Fibers, https://doi.org/10.5061/dryad.kd51c5b4w. ^24^

Data are available under the terms of the Creative Commons Zero “No rights reserved” data waiver (CC0 1.0 Public domain dedication).

## Software availability

The COMSOL software is proprietary and requires a subscription for use. The work presented in this article could instead be replicated using the open software tool FreeFEM ( https://freefem.org/).

## References

[1] L B : Diffusion de la lumière et des rayons x par un corps transparent homogène. *influence de l’agitation thermique, Annual Physic.* 1922;17:88–122. 10.1051/anphys/192209170088

[2] Vysloukh V : Nonlinear fiber optics. 1990; Vol.160.

[3] Nikles M Thévenaz L Robert PA : Simple distributed fiber sensor based on brillouin gain spectrum analysis. *Optics Letters* .1996;21(10):758–760. 10.1364/OL.21.000758 19876149

[4] Cherif R Salem AB Saini TS : Design of small core tellurite photonic crystal fiber for slow-light-based application using stimulated brillouin scattering. *Optical Engineering.* 2015;54(7):75101–75101. 10.1117/1.OE.54.7.075101

[5] Song KY Abedin KS Hotate K : Highly efficient brillouin slow and fast light using as2se3 chalcogenide fiber. *Opt Express* .2006;14(13):5860–5865. 10.1364/oe.14.005860 19516755

[6] Woodward RI Kelleher EJR Popov SV :2014.

[7] Tchahame J Beugnot J Maillotte H : Multimode brillouin scattering in a long tapered photonic crystal fiber, The European Conference on Lasers and Electro-Optics. 2015;25.

[8] Beugnot JC Lebrun S Pauliat G :2014. 10.1038/ncomms6242 PMC4220458 25341638

[9] Hu K Kabakova IV Büttner T : Low-threshold brillouin laser at 2 mm based on suspended-core chalcogenide fiber. *Opt Lett* .2014;39(16):4651–4654. 10.1364/OL.39.004651 25121840

[10] Kim M Besner S Ramier A : Shear Brillouin light scattering microscope. 2016;24(1):319–328. 10.1364/OE.24.000319 26832263 PMC4741352

[11] Rahman BMA Agrawal A : Finite Element Modeling Methods for Photonics. *Artech House.* 2013.

[12] Rahman BMA Davies JB : Finite-element solution of integrated optical waveguides. *J Lightwave Technol* .1984;2(5):682–688. 10.1007/978-1-4899-1039-4_54

[13] Poulton CG Pant R Eggleton BJ : Acoustic confinement and stimulated brillouin scattering in integrated optical waveguides. *JOSA B* .2013;30(10):2657–2664. 10.1364/JOSAB.30.002657

[14] Hughes TJ : The finite element method: linear static and dynamic finite element analysis. Courier Corporation;2012.

[15] Rahman BA Davies JB : Penalty function improvement of waveguide solution by finite elements Microwave Theory and Techniques. *IEEE Transactions.* 1984; vol.32, no.8, pp.922–928. 10.1364/JOSAA.14.001460

[16] Ham S Bathe K-J : A finite element method enriched for wave propagation problems. *Computers & Structures* .2012;94:1–12. 10.1016/j.compstruc.2012.01.001

[17] Sriratanavaree S Rahman B Leung D : Rigorous characterization of acoustic-optical interactions in silicon slot waveguides by full-vectorial finite element method. *Opt Express.* 2014;22(8):9528–9537. 10.1364/OE.22.009528 24787841

[18] Monfared V Mondali M : Semi-analytically presenting the creep strain rate and quasi shear-lag model as well as finite element method prediction of creep debonding in short fiber composites. *Materials & Design* .2014;54:368–374. 10.1016/j.matdes.2013.08.040

[19] Liu X Liu N Su X : Numerical analysis of fibers tensions in the siro-spinning triangle using finite element method. *Fibers Polymers* .2015;16(1):209–215. 10.1007/s12221-015-0209-4

[20] Lee HJ Abdullah F Emami SD : Fiber modeling and simulation of effective refractive index for tapered fiber with finite element method. *2016 IEEE 6th International Conference on Photonics (ICP), Kuching, Malaysia.* 2016; pp.1–3. 10.1109/ICP.2016.7509998

[21] Sriratanavaree S : The characterisation of acoustic waves in optical waveguides. 2014.

[22] Gan WS : New Acoustics Based on Metamaterials. 2017.

[23] Lee HJ : Modelling of Stimulated Brillouin Scattering in Graphene-clad tapered fiber using Finite Element Method.

[24] Abdul-Rashid : Dataset of Numerical Model For Enhancing Stimulated Brillouin Scattering In Optical Fibers. *DRYAD [dataset].* 2021. 10.5061/dryad.kd51c5b4w PMC10517297 37745939

