# Supplementary material for: Numerical model for enhancing stimulated Brillouin scattering in optical microfibers
Source: F1000Res. 2022 Feb 17;10:521. Originally published 2021 Jun 30. [Version 2] doi: 10.12688/f1000research.51029.2 (PMC10517297; doi:10.12688/f1000research.51029.2)
Supplement: Peer review history

# Reviewer report: Reviewer response for version 2

**DOI:** 10.5256/f1000research.120758.r199380

**Published:** 2023-09-26

**Authors:** Yu Gang Shee

**Affiliations:** 1 Centre for Photonics and Advanced Materials Research, Universiti Tunku Abdul Rahman, Selangor Darul Ehsan, Malaysia

Description on SBS: 
  Acoustic vibration across mediums scatters the incident light of the pump wave causing an **acoustic frequency shift **resulting in Stokes and anti-Stokes waves.  Please verify.
The Stokes wave propagates in the opposite direction to the pump wave.   
SBS theory was first explained by Leon Brillouin in 1922, and since then related experimental works have been performed in the past **decades**.
Consequently, at 1550 nm, a higher overlap is achieved, which contributes to a higher Brillouin gain, and therefore a lower threshold power of **1160 mW**.
  Is this correct? Please verify
5. Literature on FEM can be simplified.
"We present a numerical model to optimize Brillouin frequency shift and gain based on various core diameters of the tapered region of silica microfiber structures.'
  To optimize the modeling of Brillouin frequency shift? Or to optimize the design of fibers with desired Brillouin frequency shift/gain?
To increase/improve Brillouin gain, or Brillouin gain coefficient? Please clarify
Descriptions and discussions for Figure 3 to Figure 7 can be improved. Figures can be elaborated based on scientific justifications instead of just reporting the results.
The modeling was done on the small radius fiber. As it was claimed as the modeling for tapered fiber, is the effect of stretching section (thinning neck) of the tapered fiber is considered?

Is the rationale for developing the new method (or application) clearly explained?

Partly

Is the description of the method technically sound?

Partly

Are the conclusions about the method and its performance adequately supported by the findings presented in the article?

Partly

If any results are presented, are all the source data underlying the results available to ensure full reproducibility?

Partly

Are sufficient details provided to allow replication of the method development and its use by others?

Partly

Reviewer Expertise:

Stimulated Brillouin scattering and its applications, eg. Brillouin slow light, fiber laser

I confirm that I have read this submission and believe that I have an appropriate level of expertise to confirm that it is of an acceptable scientific standard, however I have significant reservations, as outlined above.

# Reviewer report: Reviewer response for version 2

**DOI:** 10.5256/f1000research.120758.r199375

**Published:** 2023-09-22

**Authors:** Choon Kong Lai

**Affiliations:** 1 The University of Sydney, Sydney, New South Wales, Australia

The authors provide numerical evidence demonstrating the potential enhancement of SBS gain by varying the microfiber's diameter. However, upon reviewing the manuscript, I have noted that several arguments within the paper lack precision and are inadequately described.

1. “ *Acoustic phonon scattering propagating in the backward direction*”. I find it difficult to understand this statement. Do the authors mean phonon is scattered and propagating in the backward direction? Is this true?
2. “ *Acoustic vibration across mediums scatters the incident light……transferring energy from the pump wave to the Stokes wave is known as the scattering phenomenon.*” The authors initially introduce both Stokes and anti-Stokes wave generation but subsequently concentrate solely on Stokes waves. To enhance clarity, it would be beneficial for the authors to emphasize the distinction between spontaneous and stimulated Brillouin scattering when discussing Stokes and anti-Stokes waves.
3. *“H _i_ (x, y) is the fundamental mode in optical wave*”. The *H _i_ (x, y) *should be defined as the field of the optical fundamental mode where *H ^2^* is the optical intensity. More precisely, the optical intensity should be defined in terms of both the *E* and *H* fields.
4. About Table 2, the paper does not provide a clear explanation for the sound velocity assigned to the core and clad regions. It is challenging for me to comprehend why the sound speed in these two regions needs to be adjusted differently, especially concerning cases involving shear, longitudinal, and hybrid acoustic waves. The rationale presented in the paper, specifically regarding "interruption prevention," lacks clarity and requires further elaboration. From my understanding, each bulk isotropic medium (core/cladding) possesses only one longitudinal speed and one shear speed. In this case, there will be four different speeds, *v _core,l_, v _core,s_,v _clad,l_, v _clad,s_*. Each eigenvalue returned by the solver will have different effective phase velocities and *v*_*B*_ due to different polarization fractions of shear and longitudinal components.
5. The circle diagrams in Figures 1-8 are misleading as they lack a scale reference for dimensions. Ideally, there should be three circles denoting the core, cladding, and air regions, but only two circles are presented. This omission may make it challenging for readers to discern that the inner circle represents the core, and the outer circle signifies the cladding. Additionally, the size of the simulation domain is not specified. While Dirichlet boundary conditions are mentioned, it is also crucial for the simulation domain size to be sufficiently large to ensure a close-to-zero reflection off the boundary, leading to accurate computation of effective indices, overlap ratio, and gain.
6. The absence of a color bar in all the field profiles presents a significant issue. Without a color bar representing field amplitudes, readers will struggle to differentiate between shear and longitudinal modes. Consider Figure 3 as an example, where readers could either mistakenly assume that Ux, Uy, and Uz fields are dominant, or speculate that it might represent a well-guided higher-order longitudinal acoustic mode.
7. The authors emphasize the importance of maintaining a core-clad ratio of 1:3 to prevent reflection from the outer side of the cladding. Here, “long enough” is quite subjective and it would be beneficial for the authors to explicitly clarify that this 1:3 ratio is suitable for a specific core diameter. In principle, reducing the core diameter would result in a larger mode area and, consequently, requires a larger cladding diameter. The authors must specify how the decision is being made and the range of core diameters for which the 1:3 ratio remains applicable.
8. The Brillouin gain coefficient presented in Table 6 is not specified concerning the corresponding acoustic mode (shear, hybrid, or longitudinal?). In principle, each type of acoustic mode should have a different gain coefficient.
9. In my opinion, the authors limit their presentation to just two case studies and assert that reducing the fiber diameter enhances the SBS gain of the microfiber. It would be advantageous to incorporate a broader range of fiber dimensions to better illustrate the trend they are suggesting.
10. I have identified some typos and grammar errors. For e.g.:
  - “optic propagation constant.” should be “optical propagation constant”.
  - “K _acoustic_ = 2 *β* optic.” Should be “K _acoustic_ = 2 *β*_optic_.”
  - “ *p _12_ is elasto-optic coefficient which contributes to the periodic light scattering and is FWHM of the acoustic wave in SBS.*” The Δ *v*_*B*_ is missing in this sentence.
  - “Drichhelet” should be “Dirichlet”.   

Is the rationale for developing the new method (or application) clearly explained?

Partly

Is the description of the method technically sound?

Partly

Are the conclusions about the method and its performance adequately supported by the findings presented in the article?

Partly

If any results are presented, are all the source data underlying the results available to ensure full reproducibility?

Yes

Are sufficient details provided to allow replication of the method development and its use by others?

Yes

Reviewer Expertise:

Nonlinear optics, integrated optics, Brillouin scattering

I confirm that I have read this submission and believe that I have an appropriate level of expertise to confirm that it is of an acceptable scientific standard, however I have significant reservations, as outlined above.

# Reviewer report: Reviewer response for version 1

**DOI:** 10.5256/f1000research.54134.r88748

**Published:** 2021-11-11

**Authors:** Ahmad Ashrif A Bakar

**Affiliations:** 1 Universiti Kebangsaan, Kuala Lumpur, Malaysia; 2 Department of Electrical, Electronic and Systems Engineering, Universiti Kebangsaan Malaysia, Bangi, Malaysia

This paper demonstrates the enhancement of stimulated Brillouin scattering in optical microfibers using a numerical model vectorial approach. The Brillouin shift by stimulated Brillouin scattering in a tapered silica fiber of different core and clad radii were investigated. I would suggest the authors have some amendments to improve the quality of the article. The comments are as follows.

1. Would you please put more insight into the results and discussion? The explanation of the results is too brief.
2. I'd recommend the authors to put more discussion in Figure 9 as well. It'd be interesting to see why the graphs are linear in the first stage and gradually saturated once the core diameter reaches 2μm and above.

Is the rationale for developing the new method (or application) clearly explained?

Yes

Is the description of the method technically sound?

Yes

Are the conclusions about the method and its performance adequately supported by the findings presented in the article?

Yes

If any results are presented, are all the source data underlying the results available to ensure full reproducibility?

Yes

Are sufficient details provided to allow replication of the method development and its use by others?

Yes

Reviewer Expertise:

Photonics sensing devices, surface plasmon resonance, optical feedback interferometery

I confirm that I have read this submission and believe that I have an appropriate level of expertise to confirm that it is of an acceptable scientific standard, however I have significant reservations, as outlined above.

# Response

**Published:** 2022-01-30

**Authors:** Hairul Azhar Abdul-Rashid

**Affiliations:** Multimedia University, Malaysia

Thank you for the comments made to the manuscript and questions raised. We have added to the discussion as follows: 

 Figure 9 demonstrates the relationship between *n _eff_* and silica microfiber diameter. *n _eff_* in this case here can be seen increasing as the core diameter of optical fiber increases. The *n _eff_* value increases ranging from 1.390115 to 1.446122 for 1 μm to 6 μm core diameter of the optical fiber. The values are generated based on the optical model developed in COMSOL. Based on the findings observed, the effective refractive index *n _eff_*  starts to no longer fall within the core and clad refractive index window for uniform microfibers that have core diameters 6 µm and below. This is due to the fact that modes propagating in the microfiber are no longer guided by the core and clad interface but by the air-cladding interface. It becomes more and more pronounced as the core diameter decreases.

 From the results obtained and shown in Table 4 based on the acoustic model simulations, the smaller core and clad radius of an optical fiber will produce a lower Brillouin shift frequency compared to an optical fiber with a larger core and clad radii. As for the acoustic velocities observed in the optical fibers, a smaller core and clad radii produces a higher acoustic velocity. The Brillouin Frequency shift tabulated in table 4 is obtained from the fundamental eigenvalue based on the numerical model results obtained from the COMSOL model. The Brillouin frequency shift is due to the optical-acoustic interaction. The overlap integral as stated in equation 4 demonstrates the overlap ratio between the acoustic and optical mode in the optical fiber. Shown below are the overlap ratios with respect to their core diameter. The overlap ratios are obtained by solving the overlap integral equation and are tabulated in Table 5

 From the results in Table 5, the overlap ratio is influenced by the core and cladding diameter and subsequently affect the Brillouin gain and frequency shift. This is clearly due to the optical mode and acoustic mode profile influenced by the core and cladding diameter. Based on our simulations, a typical silica uniform microfiber with parameters as mentioned above would observed a Brillouin Frequency shift around the 6GHz window.  The Brillouin frequency shift for the individual modes namely shear, longitudinal and hybrid were observed to occur at the lower end of the 6GHz spectrum for microfibers that have a smaller core and clad dimensions. As the core and clad dimensions’ increase, we observe the Brillouin Frequency shift to occur at a higher frequency in the 6GHz spectrum.  The numerical model therefore helps provide insights into fiber sensor development depending on the sensing frequency of interest. A higher acoustic velocity observed in smaller core and clad fibers also increase the overlap factor of the fundamental modes respectively. In fiber sensors, exposure to external perturbations like temperature will in effect change the acoustic velocity and the Brillouin shift frequency.

 The Brillouin gain coefficient values can be calculated by substituting the values obtained from the numerical model into the equation as denoted by Equation 5.

 Table 6 shows the Brillouin gain coefficient for the 2 optical fibers that were modeled. Between the 2 fibers modelled using the numerical model, it was observed that the optical fiber core and clad dimensions of 2 and 6 µm respectively has a higher Brillouin gain coefficient. The higher Brillouin gain coefficient is attributed to the higher overlap ratio, as shown in Table 5. One can relate this understanding to optimize the design of Brillouin amplifiers and sensors with the appropriate Brillouin gain coefficient. Thus, the numerical model here provides insights to the sensor design based on the requirements needed (25).

# Reviewer report: Reviewer response for version 1

**DOI:** 10.5256/f1000research.54134.r88747

**Published:** 2021-08-23

**Authors:** Gand Ding Peng

**Affiliations:** 1 School of Electrical Engineering and Telecommunications, University of New South Wales, Sydney, NSW, Australia

Enhancing stimulated Brillouin scattering in optical fibres is of great importance in many applications such as optical fibre sensing and optical signal amplification. In this paper, the authors presented how to numerically model SBS in silica optical fibre in terms of Brillouin frequency shift and Brillouin gain coefficient - which are the two most fundamental yet important parameters of concern.

 I read through with great interest and was helped by the fact that the paper has presented a comprehensive overview of reported work from related reference papers.

 The paper reported interesting and useful results on how fibre parameters and effective refractive index affect Brillouin frequency shift and Brillouin gain coefficient. There are issues that may need to be addressed and clarified:

 For the two cases studied, the resulted effective indexes are found to be 1.431599 and 1.441802, respectively. I note that the core and cladding refractive Indexes used in the simulation are 1.4502 and 1.445, respectively. Normally we would expect that the effective index of optical mode to be within core and cladding indexes, i.e. between 1.4502 and 1.445. Why both the effective indexes are lower than 1.445 here?

 In describing the method to obtain Brillouin frequency shift and gain coefficient, it is not clear how the optical - acoustic wave overlap factor (equation 4) is related to the Brillouin gain coefficient (equation 5). A simple explanation would be helpful. In addition, It would be helpful to explain how the Brillouin frequency shift is determined.

 It is not very clear how the values of sheer and longitudinal velocities are assigned for core and clad for different cases. For example, it is stated that ‘in the case of pure shear acoustic, longitudinal velocity for the core region were made equivalent to 5736 m/s, this is to prevent interruption of longitudinal acoustic wave’. Please explain what ‘interruption’ means here.

 (Please note: the actual value for longitudinal acoustic wave velocity in the shear acoustic case in Table 2 is 5933, not 5736 as stated.)

 In the abstract, it is stated that ‘This paper describes the method related to the numerical model and discusses the analysis between the interactions of horizontal, shear and hybrid acoustic modes; and optical modes in optical fiber’. But the rest of the paper ‘horizontal’ is replaced with ‘longitudinal’. It would be better to keep consistency. In addition, more analysis/discussion of the interaction of these acoustic modes would be helpful.

 In this paper, it frequently referred to the two cases of different core sizes (with the same core/cladding ratio) as tapered fibre. This is not really tapered fibre cases anyway. They are just two straight optical fibre cases. The use of tapered fibre is a bit confusing. For example, in the conclusion, it is stated that ‘The Brillouin Frequency Shift vB occurs at lower frequencies for a

 tapered fiber with smaller core and cladding dimensions’. Here ‘for a tapered fiber’  seems inaccurate since the results are based on numerical simulation of a uniform fibre case.

 In the paper, it has used ‘Brillouin gain coefficient’ and ‘Brillouin gain’ alternatively. It must be noted that they are two distinctive parameters with different units normally. For example, in the abstract, it is mentioned as ‘gain’ in the statement ‘Based on this numerical model, we report the influence of core radius, clad radius, and effective refractive index on the Brillouin frequency shift and gain’. Similarly, Table 6 should be ‘Brillouin gain coefficient’ and ‘Brillouin gain’. Perhaps ‘gain coefficient’, instead of 'Brillouin gain', is a slightly better alternative for ‘Brillouin gain coefficient’ if needed. 

 Some of the references didn’t include the sources. For example, refs 21-23.

 Finally, the authors may check spelling and wording for better clarity For example, it is stated in the abstract that ‘SBS was previously viewed as a poor method due to the limitation on optical power in high-powered photonic applications’. It seems to me that ‘method’ is not an appropriate term here.

 In summary, although there are aspects that the paper could be improved upon as mentioned above, the paper presented an important work of interest to people in the field and is worthy of indexing and further working on.

Is the rationale for developing the new method (or application) clearly explained?

Yes

Is the description of the method technically sound?

Yes

Are the conclusions about the method and its performance adequately supported by the findings presented in the article?

Yes

If any results are presented, are all the source data underlying the results available to ensure full reproducibility?

Partly

Are sufficient details provided to allow replication of the method development and its use by others?

Partly

Reviewer Expertise:

Photonics and Optical Fibres

I confirm that I have read this submission and believe that I have an appropriate level of expertise to confirm that it is of an acceptable scientific standard.
